# A systematic review of adherence to iron chelation therapy among children and adolescents with thalassemia

**DOI:** 10.1080/07853890.2022.2028894

**Published:** 2022-02-01

**Authors:** Paavani S. Reddy, Margaret Locke, Sherif M. Badawy

**Affiliations:** a Department of Medical Education, Northwestern University Feinberg School of Medicine, Chicago, IL, USA; b Department of Internal Medicine, Zucker School of Medicine at Hofstra/Northwell Institute, Hempstead, NY, USA; c Department of Pediatrics, Northwestern University Feinberg School of Medicine, Chicago, IL, USA; d Division of Hematology, Oncology, and Stem Cell Transplant, Ann & Robert H. Lurie Children’s Hospital of Chicago, Chicago, IL, USA

**Keywords:** Thalassaemia, iron chelation therapy, adherence, compliance, interventions, behaviour

## Abstract

**Introduction:**

Iron chelation therapy (ICT) is essential to prevent complications of iron overload in patients with transfusion-dependent thalassaemia. However, there is currently no standard for how to best measure adherence to ICT, nor what level of adherence necessitates concern for poor outcomes, especially in paediatric patients. The objectives of this review are to identify rates of adherence to ICT, predictors of adherence, methods of measurement, and adherence-related health outcomes in children and adolescents.

**Methods:**

This review covers the literature published between 1980 and 2020 on ICT in thalassaemia that assessed adherence or compliance. Included studies reflect original research. The preferred reporting items of systematic reviews and meta-analyses (PRISMA) guidelines were followed for reporting results, and the findings were critically appraised with the Oxford Centre for Evidence-based Medicine criteria.

**Results:**

Of the 543 articles, 37 met the inclusion criteria. The most common methods of assessing adherence included patient self-report (*n* = 15/36, 41.7%), and pill count (*n* = 15/36, 41.7%), followed by subcutaneous medication monitoring (5/36, 13.8%) and prescription refills (*n* = 4/36, 11.1%). Study sizes ranged from 7 to 1115 participants. Studies reported adherence either in “categories” with different levels of adherence (*n* = 29) or “quantitatively” as a percentage of medication taken out of those prescribed (*n* = 7). Quantitatively, the percentage of adherence varied from 57% to 98.4% with a median of 89.5%. Five studies focussed on interventions, four of which were designed to improve adherence. Studies varied in sample size and methods of assessment, which prohibited performing a meta-analysis.

**Conclusions:**

Due to a lack of clinical consensus on how adherence is defined, it is difficult to compare adherence to ICT in different studies. Future studies should be aimed at creating guidelines for assessing adherence and identifying suboptimal adherence. These future efforts will be crucial in informing evidence-based interventions to improve adherence and health outcomes in thalassaemia patients.

**Key messages**
- Predictive factors associated with ICT adherence in the paediatric population include age, social perception of ICT, social support, and side effects/discomfort.
- Increased adherence in the paediatric population is associated with decreased serum ferritin and improved cardiac, hepatic, and endocrine outcomes.
- Inadequate adherence to ICT is associated with increased lifetime health costs.
- There are few studies that focussed on interventions to increase adherence in the paediatric population, and the studies that do exist all focussed on different types of interventions; successful interventions focussed on consistent, long-term engagement with patients.

## Introduction

Thalassaemia is a common inherited haemoglobin disorder characterized by reduced or absent production of beta globin chains, leading to destruction of red blood cells and chronic anaemia. One of the mainstays of treatment for thalassaemia is regular packed red blood cell (pRBC) transfusions. However, transfusions can lead to excess systemic iron overload with accumulation of iron in the heart, liver, spleen, and other tissues, which can lead to a wide array of complications. These complications include endocrinopathies, cardiomyopathy, and hepatic failure. Iron overload is the major cause of morbidity and mortality in thalassaemia [1]. Thus, managing post-transfusional iron overload with iron chelation therapy is very critical.

There are three main iron chelation agents including deferoxamine (DFO), deferiprone (DFP), and deferasirox (DFX). DFO must be administered subcutaneously or intravenously up to once a day due to poor oral bioavailability; DFP and DFX may be administered orally up to three times a day [2]. Known side effects include infusion reactions in DFO, agranulocytosis in DFP, gastrointestinal distress and transaminitis in DFP and DFX [2]. Prior studies have suggested that these side effects, as well as the inconvenience of parenteral administration of DFO and frequency of DFP and DFX administration, lead to reduced adherence to iron chelation regimens, and subsequently, poorer control over iron deposition in vital organs [3,4]. Such studies highlight that difference chelators may have difference causes of non-adherence due to variation in side effects and route of administration. Moreover, DFX may be available in both dispersible tablet (DT) form and as a film coated tablet (FCT); one study found that patients consistently found DFX (FCT) more palatable than DFX (DT), as patients experienced less gastrointestinal adverse events [5]. Studies have shown differences in chelators’ ability to control iron load, such as the superiority of DFP in reducing myocardial iron load and DFO in reducing hepatic iron load [4]. Although data is limited, a recent cost analysis estimated that inadequate adherence to iron chelation regimens leads to numerous complications and associated lifetime costs of $33,142 [3].

Currently, the international guidelines of which chelator or combination of chelators to take vary widely and depend partially on local practice guidelines, clinical judgement, patient age, individual patient’s iron profile, iron intake, as well as the amount of cardiac and liver iron deposition and presence or absence of heart failure. However, decisions for which combination of iron chelators to implement must also depend on the efficacy and safety of the chelation regimen with the likelihood of patient adherence to the regimen. Previous work has shown that adherence varies widely even between iron chelators. In prior studies of parenteral deferoxamine, mean adherence ranged from 59 to 78%, whereas adherence to oral deferiprone reported higher adherence, between 79 and 98% [3]. This disparity in adherence is evidence that factors such as inconvenience, painful administration, and side effects all factor into the rate of adherence to iron chelation therapy ultimately affecting health-related patient outcomes and quality of life [6]. These factors are also important when considering combination therapy, due to the combination of these barriers with potentially different routes of administration and side effects, in addition to a more strenuous medication regimen for patients. Quantification of adherence to each regimen as well as patient psychosocial characteristics, socioeconomic status, and other factors that may affect adherence are important to elucidate. This will help determine which regimen best balances chelation efficacy with patient adherence as well as potential strategies to improve patient adherence to iron chelation therapy.

Despite this, there is very little consensus on what defines appropriate adherence to iron chelation therapy or how best to measure it. Several known and validated methods include the Morisky Medication Adherence Scale [7] (MMAS), Visual Analog Scale [8] (VAS), pill bottle review, including the Medication Event Monitoring System [9] (MEMS), and clinician gestalt of the patient interview. This leads to high variability in reported adherence as well as varying definitions of what constitutes adequate adherence to iron chelation therapy. Recent reviews have estimated that general medication adherence in paediatric populations with chronic health conditions is between 50 and 75 percent, especially with the lowest rates in adolescents [10]. Several factors that affect medication adherence in adolescents include social and schedule-related pressures as evidenced by poor weekend adherence compared to weekdays [11], medication-related factors such as complexity of the regimen and side effects, and personal factors such as forgetfulness and health literacy [12]. These highly variable forms of measurement as well as the lack of knowledge about specific paediatric and parent factors that could impact adherence make it hard for physicians to quantify what degree of adherence they can expect from their patients or identify concrete areas for change to improve adherence.

The objectives of this systematic review are to evaluate adherence rates to iron chelation therapy among children and adolescents with thalassaemia, assess methods of measurement, predictors of adherence, and adherence-related health outcomes. This presents an opportunity to unify the methodologies used to measure adherence as well as to assess how factors such as drug delivery routes, adverse outcomes, or other factors may affect adherence rates in children and adolescents with thalassaemia.

## Methods

### Search strategy

A librarian developed highly sensitive medical subject headings (MeSH) term‒based search strategy collaboratively with other review authors in May 2018 searching in the following databases: PubMed MEDLINE; Embase; Cochrane Central Register of Controlled Trials (CENTRAL) on the Wiley platform; the Cumulative Index to Nursing and Allied Health Literature (CINAHL) (EBSCO); PsycINFO (EBSCO). Search strategies for all databases except MEDLINE were adapted from the PubMed MEDLINE strategy. Additional searches of PubMed were run in November 2020. All databases were searched back to 1980, which is a point in time when deferoxamine and other iron chelators began being used more widely in the clinical treatment of iron overload. No language limits were applied. The search strategy specified keywords, including iron chelation, iron chelator, desferrioxamine, deferoxamine, deferasirox, alpha or beta thalassaemia, children, paediatric, adolescent, youth, and adults. We also attempted to identify additional studies by searching the reference lists of key studies and relevant systematic reviews. We contacted the authors of the included publications to obtain additional studies meeting the inclusion criteria. Two independent reviewers (ML and SB) assessed abstracts and articles against eligibility criteria and critically appraised the methodological quality. Disagreements were resolved by discussion or consultation with a colleague, if needed.

### Study selection

The inclusion criteria were as follows: (1) children with alpha or beta thalassaemia requiring iron chelation therapy, (2) measured adherence or compliance to iron chelation therapy, (3) original research articles, (4) studies that were either randomized controlled trials, quasi-experimental studies, or pilot/feasibility studies (including single arm, pre-posttest), (4) text messaging or mobile phone‒based interventions (app or mobile intervention), and (5) medication adherence as the primary or secondary outcome.

The exclusion criteria were as follows: (1) limited or no assessment of adherence or compliance, (2) no definition of adherence or compliance that was assessed, (3) case reports, (4) no English version available, (5) study population without thalassaemia, (6) mean patient age >18 years

### Data extraction

We used a standardized form for data extraction. Data items in the extraction form included the following: first author’s name; journal; publication year; country; type of thalassaemia at the focus of the study; sample size; participants’ age; sex; recruitment method; study design; duration of intervention and follow-up; inclusion criteria; exclusion criteria; adherence measurement methodology; adherence definition; adherence rates; predictors of adherence; other outcome measures such as disease-related outcomes of morbidity and mortality, HRQOL, and self-efficacy or self-management skills; and theoretical framework. Two authors (ML and SB) coded all included articles individually, and then the lead author independently reviewed all codes. Disagreements were resolved by discussion or by consultation with a third author, if needed.

### Data analysis

Data were analyzed quantitatively and qualitatively. Our primary outcome measure was mean iron chelation therapy adherence rate as well as the common methodology used to assess adherence rates. Additional outcomes included predictors of adherence and adherence-related outcomes such as serum ferritin levels and psychosocial outcomes.

## Results

### Literature search

**Figure 1.**
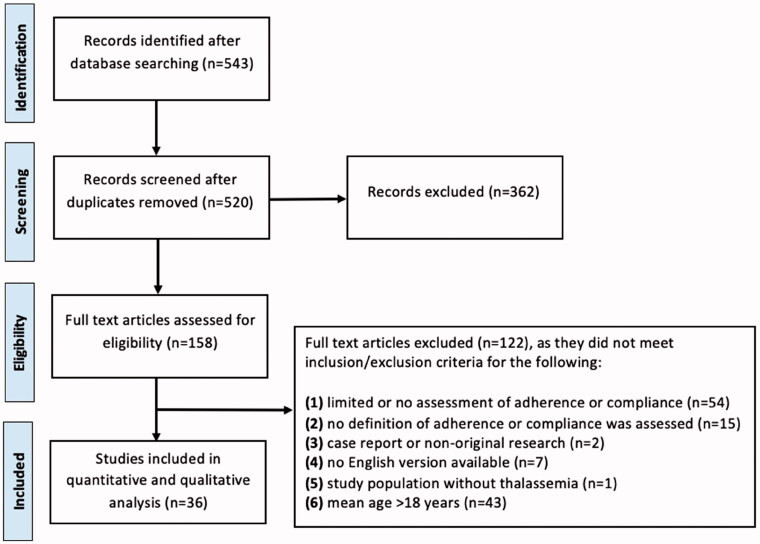
Prisma guidelines included/excluded articles.

The literature search identified 543 articles. 36 articles met all inclusion and exclusion criteria (Figure 1).

### Methodological quality

**Figure 2.**
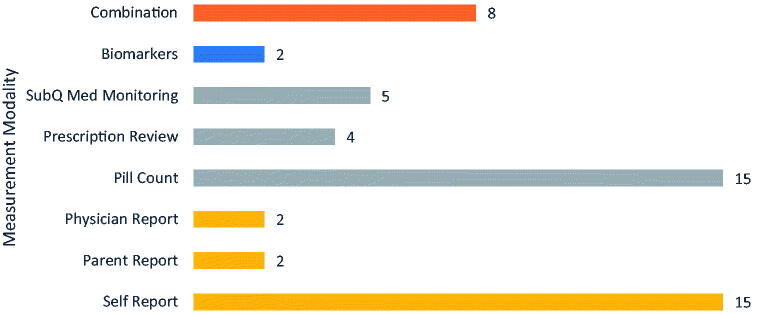
Methods of adherence measurement. *Combination includes studies in other subgroups*.

All studies (*n* = 36) recruited entirely through the clinic. Some studies also focused on specific chelators, or compared chelators with one another: 11 studies focused on DFX, 17 on DFO, and 6 on DFP, with 3 articles focusing on some combination therapy (3/36, 8.3%), although none providing a direct comparison of adherence rates for combination versus single therapy. Main adherence outcomes are summarized in Table 2. Studies focusing on DFX did not distinguish between DT or FCT formulation. Of the 36 articles, five focused on a specific intervention designed to improve adherence (Table 3). This focus of this paper, adherence, was measured in a variety of ways across all papers, including patient self-report, pill count, prescription refill history, subjective scoring by the physician/provider, and a combination of the aforementioned techniques. Several studies used some system of individual reporting, whether self-report (15/36, 41.6%), parent report (2/36, 5.6%), or physician/provider report (2/36, 5.6%). Other studies used some form of medication monitoring, whether through pill count (15/36, 41.6%), prescription refill history (4/36, 11.1%), or subcutaneous medication monitoring (5/36, 13.5%). Two studies used biomarkers as a measurement of adherence (2/36, 5.6%), A few studies (8/36, 22.2%) used multiple methods to cross-reference level of adherence the patients had with ICT, and if needed, would prioritize one method over another [14–21]. Figure 2 summarizes different approaches for measuring adherence in the included studies.

### Description of included studies

Table 1 summarizes the study characteristics. Seven studies were conducted in the United States and Egypt, three in Italy and Jordan, 2 in Lebanon, Malaysia, Turkey Syria and Oman, and one in Thailand, Canada, UK, Greece, Brazil, Pakistan, Taiwan, and Saudi Arabia. Of the thirty-seven studies, one of them were conducted in multiple countries [43].

**Table 1.** Summary of included studies focussed on iron chelation adherence.

Source (country)	Study objective	Study design follow-up duration	Type of thalassaemia	Sample size (*n*)	Age (SD, range)*	Sex (% female)
Abd (2017) [13], Iraq	To examine the benefits and side effects of Deferasirox in thalassaemia patients	Cross-Sectional 24 months	All thalassaemia major	50	_*33 patient*s* <6 years old, 17 patient*s* >6 years old	50
Aina Mariana (2014) [14], Malaysia	To evaluate management , clinical outcomes of transfusion dependent children at an ambulatory care centre relative to Malaysian Clinical Practice Guidelines	Cross-Sectional 3 months	All thalassaemia major	26	(2–15)	46.2
Al-Kloub (2014) [15], Jordan	To examine relationship between psychosocial status, disease knowledge, adherence to DFX in adolescents with thal major	Cross-Sectional 4 months	All thalassaemia major	36	17.5 (2.03, 12–19)	61
Al-Kloub (2014) [16], Jordan	To identify rates and predictors of non-adherence to follow-up visits and Deferasirox chelation therapy	Cross-Sectional 4 months	Beta thalassaemia	124	15.06 (2.28, 12–19)	49
Arboretti (2001) [17], Italy	To determine whether deferoxamine is better tolerated and improves compliance compared to parenteral iron chelation therapy	Cross-Sectional 9 months	All thalassaemia	867	17.8 (2–50.3)	52
Aydinok (2005) [18], Turkey	To evaluate the psychosocial burden and medication compliance in thalassaemia patients	Cross-Sectional	All thalassaemia major	38	12.2 (3.3, 6–18)	52.6
Bahnasawy (2017) [19], Egypt	To determine the impact of clinical pharmacist-provided services on the outcome of iron-overloaded BTM children	RCT 9 months	Beta thalassaemia major	48	12.52	62.50
Beratis (1989) [20], Greece	To determine non-compliance to deferoxamine, as well as contributing psychological factors.	Case Control	All thalassaemia major	113	15.2	53.9
Boturao-Neto (2002) [21], Brazil	To identify non-invasive methods to evaluate the severity of iron overload in transfusion-dependent ß-thalassaemia patients and to assess the efficiency of intensive intravenous therapy	Cross-Sectional	All thalassaemia major	26	14.7 (4–50)	46.1
Canatan (1999) [22], Turkey	To determine compliance and side effects to two different DFO infusers: group A (48 hr), and group B (120 hr)	RCT	All thalassaemia major	26	16.1 (4, 11–29)	Not reported
Capra (1983) [23], Italy	To examine the difference in compliance between patients with diabetes or impaired glucose tolerance normal tolerance	Cross-Sectional 36 months	All thalassaemia	60	15.2 (3.4, 9–23)	41.57
Daar (2006) [24], Oman	To compare DFX vs. DFX and L1 combined therapy	Cohort Study	Beta thalassaemia major	55	15.02 (5.8, 2–30)	54.90
De Sanctis (2006) [25], Italy	To determine the association between endocrine, hematological symptoms and compliance	Cross-Sectional	All thalassaemia major	238	12.7 (4.2, 1.9–19.6)	47.50
Elalfy (2012) [26], Egypt	To examine the development, validation of the ICT-SAT tool for treatment satisfaction	Cross-Sectional	Beta thalassaemia major	152	12.7 (7.3, 2–31)	46.1
Elalfy (2013) [27], Egypt	To determine if Hepatitis C infection is related to liver fibrosis and compliance in thalassaemia patients	Cross-Sectional	Beta thalassaemia	51	15.9 (3.1, 12–24)	49
Elalfy (2015) [28], Egypt	To compare DFX + DFP vs DFO + DFP in relation to adverse effects, iron overload	RCT	Beta thalassaemia major	96	DFO + DFP: 15.3 (2.3) DFO + DFX: 14.1 (2.2)	DFO + DFP: 37.5, DFO + DFX: 33.4
El-Beshawy (2008) [29], Egypt	To compare DFP + DFO vs. DFP vs. DFO	RCT	All thalassaemia major	66	DFP + DFO: 11 (4.9) DFP: 10.8 (5.1) DFO: 13.1 (5.9) Overall Range: 5–26	45.45
Keikhaei (2011) [30], Pakistan	To assess the efficacy and safety of the sequential DFO and DFX/OSV protocol, and the combination of Deferoxamine/ Deferiprone in thalassaemia major patients	RCT	Beta thalassaemia major	290	DFO + DFP: 17 (2–36) Seq OSV + DFO: 18.5 (6–30)	DFO + DFP**:** 48 Seq OSV + DFO: 50
Koch (1993) [31], USA	To determine the efficacy of a contingency and behavioural monitoring program designs to help patients increase or maintain their use of deferoxamine	Cohort Study 12 months	Beta thalassaemia major	23	14.7 (3–30)	69
Lai (2013) [32], China	To assess the efficacy and safety of Deferasirox in Chinese thalassaemia major patients vs non-Chinese patients	Cohort Study	All thalassaemia major	1115	Chinese: 6.8 (2–19) Non–Chinese: 19.5 (2–72)	Chinese: 40 Non-Chinese: 53
Lee (2009) [33], Taiwan	To examine disease knowledge in thalassaemia major patients and their mothers and to understand the relationships between disease knowledge and treatment adherence.	Cross-Sectional	Beta thalassaemia major	32 patients**32 parents	Patients: 17.5 (12–29) Parents: 40.5	52
Lee (2011) [34], Malaysia	To analyse the self reported degree of compliance, as well as the factors influencing compliance to DFO therapy in children with transfusion dependent thalassaemia major in Malaysia	Cross-Sectional	All thalassaemia major	139	Median 9 (2–16)	47
Leonard (2017) [35], USA	To determine the feasibility of an “intensive training program” ITP, developed by the authors and its preliminary impact on adherence, disease knowledge, and health outcomes	Cohort Study	Beta thalassaemia major	11	12.4 (3.8)	64
Lerner (1990) [36], USA	To determine the effect of iron chelation medication o the cardiac status of older patients	Cohort Study 4 years	Beta thalassaemia major, beta thalassaemia intermedia	10	17.5 (12–24)	Not reported
Maurer (1998) [37], USA	To examine the results of iron chelation therapy on paediatric patients	Cohort Study	Beta thalassaemia	16	(3–17)	Not reported
Mokhtar (2013) [38], Egypt	To assess the morbidity and mortality of transfusion dependent thalassaemia patients, and compare the outcomes in relation to age of onset, type of medication, duration, and compliance	Cross-Sectional 10 years	Beta thalassaemia	477	14.2 (7.7, 9–35)	36.6
Olivieri (1991) [39], Canada	To determine whether MEMS a good system of measurement for medication compliance	Cross-Sectional	Beta thalassaemia	7	17.4 (10–22)	Not reported
Payne (2007) [40], USA	To determine the burden of infused ICT conducted in four US centres	Cohort Study	Beta thalassaemia	49	(6–21)	49
Payne (2008) [41], UK	To examine clinical, health related quality of life, and economic outcomes associated with iron chelation therapy	Cohort Study	Beta thalassaemia	60	(6–19)	63
Shosha (2019) [42], Jordan	To assess beliefs and adherence associated with both oral deferasirox and deferoxamine infusion chelation therapy among Jordanian children with Thalassaemia major, and compare the adherence levels between recipients of each	Cross-Sectional	All Thalassaemia Major	120	13.7 (8–18)	57.6
Taher (2010) [43], Multi-institutional**	To investigate patient reported outcomes of once-daily oral Deferasirox with prior DFO and DFP use.	Cohort Study	Beta thalassaemia	237	13.3 (7.1, 2–42)	49.4
Treadwell (2011) [44], USA	To determine behavioural aspects of compliant with DFO, to explore social factors that might influence compliance, to evaluate the effectiveness of an intervention program.	RCT	Beta thalassaemia	31	SCD: 11.6 (4.6) Thalassaemia: 11.9 (4.7)	51.61
Viprakasit (2013) [45], Thailand	To determine the clinical efficacy and safety of DFP	Cohort Study	Beta thalassaemia	73	(3.2–19)	Not reported
Wolfe (1985) [46], USA	To examine the efficacy of long term subcutaneous DFO therapy	Cohort Study 6 years	Beta thalassaemia major	36	12.4 (3, 10–28)	Not reported
Yassouf (2019) [47], Syria	To identify the existence of hypothyroidism in patients with beta-thalassaemia in Syria, and evaluate the effect of compliance with DFO on thyroid function	Cross-Sectional	Beta thalassaemia major	82	17.5 ** (10–25)	51.20
Zahed (2002) [48], Lebanon	To evaluate the reactions of patients to new oral chelation therapy	Cross-Sectional	Beta thalassaemia major, beta thalassaemia intermedia	44	19** (12–38)	39

*Age was reported as Mean (SD, Min–Max) unless otherwise stated.

**Institutions included in Taher (2010): Egypt, Lebanon, Oman, Saudi, Arabia, Syria.

**Table 2.** Main adherence outcomes of included studies.

Source (country)	Adherence outcomes Methodology Categorical or quantitative, criteria Adherence statistics	Other study outcomes
Abd (2017) [39], Iraq	Parent report Categorical good vs. poor 70% good	Serum ferritin– decreased from 2678.83 ng/ml before Deferasirox to 2255.43 ng/ml after Deferasirox Gastrointestinal symptoms in 12% Skin rash in 2% Liver enzyme increase in 4%
Aina Mariana (2014) [34], Malaysia	Pill count Categorical compliant vs. noncompliant DFO: 47% compliant DFP: 90% compliant DFX 66.7% compliant	Lower adherence associated with: ○In DFO users: ○Unreliable schedules ○Family (reliance on one parent for injection) ○Disease severity (increased pain) ○In DFO users: ○Forgetfulness ○In DFX users: ○Dislike of medication 21 Patients were found to have a serum ferritin of more than 1000 μg/L, despite chelation with optimum dosage
Al-Kloub (2014) [14], Jordan	Patient self-report, biomarker (serum ferritin) Categorical 14% Full 31% Partial 56% Low	Lower adherence associated with: ○Age (patient*s* < 16 years) ○Family (1 or more siblings with thalassaemia) ○SES (lower family income)
Al-Kloub (2014) [40], Jordan	Patient self-report Categorical 73% Full 21% Partial 6% Poor	Lower adherence associated with: ○Age (adolescents, patient*s* > 16 years) ○Family (1 or more siblings with thalassaemia, lack of parental involvement) ○Psychosocial impairment Serum ferritin– only 62.1% of participants achieved the recommended serum ferritin level (≤2500 μg/L),
Arboretti (2001) [41], Italy	Subcutaneous treatment completed vs expected Categorical 64% Good 27% Fair 9% Poor	Higher adherence associated with ○Quality of life (increased perception of quality of care, decreased sense of discomfort)
Aydinok (2005) [42], Turkey	Subcutaneous treatment completed vs. expected Categorical 47% Compliant 53% Noncompliant	Lower adherence associated with: ○Family (1 or more siblings with thalassaemia, “familial issues”) ○Disease severity– increased disease complications Higher adherence associated with ○Psychological issues (increased anxiety, depression, internalizing problems)
Bahnasawy (2017) [32], Egypt	Patient self-report Categorical 73% Full 21% Partial 6% Poor	Lower adherence associated with: ○Increased thalassaemia complications Higher adherence associated with ○Healthcare satisfaction (increased) ○Quality of life (increased)
Beratis (1989) [26], Greece	Patient self-report Categorical (Compliant vs. non-compliant Non-compliant: <60% of recommended dose) 77% cOmpliant 23% Noncompliant	Lower adherence associated with: ○Psychological (increased psychiatric disorders) ○Family (increased familial and social issues) Unrelated to compliance ○Age of ICT initiation ○Number of siblings
Boturao-Neto (2002) [43], Brazil	Subcutaneous treatment completed vs expected Categorical (Compliant vs. non-compliant Non-compliant: <80% of treatments completed) 65.4% Compliant 34.6% Noncompliant	Lower adherence associated with: ○Age (older age) ○Serum ferritin (higher levels) ○Cardiovascular health (increased cardiac abnormalities)
Canatan (1999) [44], Turkey	Subcutaneous treatment completed vs. expected Quantitative 48 h DFO: 97% compliant 120 h DFO: 72% compliant	A 48 h DFO regimen is associated with better compliance and fewer complications
Capra (1983) [22], Italy	Patient self-report Categorical Regular: 6+ days/week Irregular: 3–5 days/week None: <3 days/week 16.67% Regular 43.5% Irregular 39.7% None	Compliance was found to be significantly lower in diabetic patients
Daar (2006) [45], Oman	Prescription refill history Categorical 61.8% Compliant 38.2% Noncompliant	Higher adherence associated with ○Cardiovascular health (improved cardiac ejection fraction)
De Sanctis (2006) [15], Italy	Pill count, physician/provider rating, biomarkers (serum ferritin) Categorical Good vs. poor Good: 4+ days/week OR serum ferritin <2500 ug/L 63% Good 37% Poor	
Elalfy (2012) [46], Egypt	Physician/provider rating Categorical Poor vs. good vs. excellent Poor: <50% Good: 50–80% Excellent: >85% 32.9% Poor 55.3% Good 9.9% Excellent	Compliance was associated with perceived effectiveness, the patient’s sense of fear, and the severity of side effects of the medications Serum ferritin levels correlated with adherence
Elalfy (2013) [16], Egypt	Patient self-report, pill count Categorical Group 1: 11% compliant Group 2: 31% compliant Group 3: 73% compliant Total: 49% compliant, 51% noncompliant	Lower adherence associated with: ○Liver health– HCV positivity and LI*C* > 14 mg/g High adherence associated with: ○Age of ICT initiation ○Number of siblings
Elalfy (2015) [28], Egypt	Pill count Quantitative Group A: 95% compliance Group B: 80% compliance	Higher adherence associated with: ○Oral iron chelators
El-Beshawy (2008) [47], Egypt	Pill count Categorical 94% compliant 6% noncompliant	
Keikhaei (2011) [17], Pakistan	Patient self-report, pill count Quantitative DFO + DEF: 93% compliance SeqOSV + DFO: 95% compliance	High adherence associated with: ○Lower medication side effects Serum ferritin– ○DFO + DEF: serum ferritin declined from 2564.69 to 2050.44, ○SeqDFO/OSV: serum ferritin declined from 3590 to 2563
Koch (1993) [25], USA	Pill count Categorical Good- 100% pills taken Bad- less than 100% 69% Good	High adherence associated with: ○Psychosocial (positive reinforcement through careful monitoring and a behavioural reward system
Lai (2013) [18], China	Prescription refill history, pill count Categorical Compliant vs non-compliant Compliant >80% pills taken 92% Compliant 8% Noncompliant	Unrelated to compliance ○Age ○Ethnicity ○Oral chelation medications Serum ferritin–levels less than 2000 were observed in 91.5% of Chinese patients, and 74.5% of non-Chinese patients.
Lee (2009) [48], Taiwan	Subcutaneous treatment completed vs expected Categorical Full vs. partial vs. poor 48.4% Full adherence 32.2 Partial adherence 19.4% Poor adherence	High adherence associated with: ○Disease knowledge about thalassaemia major
Lee (2011) [29], Malaysia	Patient self-report Categorical Not compliant vs. poorly compliant vs. Moderately compliant vs. very compliant 31% Very compliant 50% Moderately compliant 3% Poorly compliant 16% Not compliant	High adherence associated with: ○Oral chelator medications Serum ferritin– serum ferritin was high for this study group, because of short duration of DFO therapy and compliance
Leonard (2017) [33], USA	Pill count via application Quantitative 58% compliance	High adherence associated with: ○Mobile app use (mobile ITP app with reminders) ○Disease knowledge retention
Lerner (1990) [23], USA	Patient self-report Categorical Compliant vs noncompliant Compliant: 5 or more days/week 58% Compliant	Lower adherence associated with: ○Cardiovascular health (more likely to develop cardiac issues and die) High adherence associated with: ○Oral iron chelator medications Serum ferritin– 8 out of the 10 patients had decrease in serum ferritin levels, 2 had increases
Maurer (1998) [24], USA	Prescription refill history Categorical Compliant vs. noncompliant Compliant: 5 or more days/week 68.75% Compliant	
Mokhtar (2013) [49], Egypt	Pill count Categorical Compliant vs noncompliant DFO: 82.3% compliant DFX: 100% compliant DFP: 92.2% compliant	High adherence associated with: ○DFX over other iron chelators
Olivieri (1991) [31], Canada	Pill count Categorical Compliant vs noncompliant Compliance is >95.7% doses taken 88.7% Compliance	
Payne (2007) [50], USA	Pill count Categorical Low compliance: 0–50% Partial compliance: 51–80% High compliance: 81+% 23% Low compliance 36% Partial compliance 41% High compliance	Lower adherence associated with: ○Disliking the mode of administration of medication ○Side effects (increased) ○High serum ferritin levels Cost– the total cost of ICT for the patient appears to exceed the drug cost.
Payne (2008) [35], UK	Patient self-report Categorical Over the course of 1 week: 50% Missed one dose 46% Missed two or more doses Over the course of 4 weeks: 77% Missed one dose	Lower adherence associated with: ○Adverse health events (14% of patients who missed a dose did so due to adverse events) ○Serum ferritin (higher levels with less compliance) Serum ferritin ○Mean ferritin level was 3615+/-3522 ng per mL when less than 50% compliant ○Mean ferritin level was 2831+/-2474 ng per mL when between 51 and 80% compliant, ○Mean ferritin level was 1573+/-1694 ng per mL when more than 80 percent compliant. Cost– mean weighted costs of medications is 3671 pounds for patients, but cost increases to 4421 pounds when 100% compliance is assumed.
Shosha (2019) [30], Jordan	Patient self-report using MARS-5 Categorical Compliant when rarely/never skipped a dose DFO: 89.8% compliant DFX: 92.2% compliant Average MARS-5 score: 23.44	Lower adherence associated with: ○Age (younger ages– adherence from children 8–12 were higher than children older than 12 with both medications) Oral chelators had a higher adherence rate, but this was not statistically significant.
Taher (2010) [13], Multi-institutional	Pill count Quantitative 98.4 +/-4.6% overall compliance 98.7+/-3.2% paediatric compliance 97.9+/-0.7% adult compliance	Oral chelators provided patient centred benefits, reflected in high persistence and compliance rate. Deferasirox treatment was efficacious- the cohort had overall decreases in liver iron concentration (-3.4 mg Fe/g dry weight) and serum ferritin (-341 ng/mL) over 1 year of treatment
Treadwell [19] (2011), USA	Patient self-report, parent report Quantitative Initial: 57% compliance Midpoint: 83% compliance Ending: 73% compliance	Higher adherence associated with: ○Psychosocial (perceived support) ○Knowledge (caregiver/patient knowledge) Compliance did not change after the intervention
Viprakasit (2013) [20], Thailand	Patient self-report, pill count Quantitative 94.48 +/-6.06%	Lower adherence associated with: ○Length of treatment (the longer a patient was on a new treatment plan, the lower the adherence) High adherence associated with: ○DFP use over DFO use Serum ferritin– ○Mean serum ferritin levels at 1 year were not significantly changed from baseline. ○45% of patients had SF reduced 15% from baseline at 1 year, with a median reduction of 1,065 ng/mL
Wolfe (1985) [21], USA	Patient self-report, prescription refill history Categorical Considered compliant when remembered doses 5 or more days/week 47.22% Compliant	High adherence associated with: ○Cardiovascular health (Improved– 1 of the 17 patients in the compliant group had cardiac disease, 12 of the 19 is in the noncompliant group) Decreased serum ferritin Serum ferritin– ○The mean ferritin level in the compliant group fell from 4765 +/-610 to 2950 +/-1850 ng per millimetre, ○The ferritin level of the noncompliant group rose from 5000 +/-2316 to 6040+/-550 ng per millimetre.
Yassouf (2019) [51], Syria	Pill count using medication possession ration (MPR) Categorical Considered adherent if MPO >0.80 54.87% Compliant	Hypothyroid patients had an average medication possession ratio of 40.2%, euthyroid patients had a medication possession ratio of 68.51%
Zahed (2002) [27], Lebanon	Patient self-report Categorical Full compliance vs. Irregular compliance vs. No compliance 16% Full compliance 45% Irregular compliance 39% No compliance	High adherence associated with: ○Oral chelator medication (oral ICT therapy was associated with higher compliance, except in one patient who had adverse side effects– ^severe arthralgia and nausea) Preference for DFP was associated with: ○Psychological relief in 55% of cases ○Relief from DFO pump in 27% of cases ○Financial relief in 9% of cases 9% of patients were indifferent to DFP treatment Cost– deferiprone is less expensive ($3000 per patient per year) compared to desferrioxamine ($9000)

### Adherence levels

Measures of adherence were reported in two studies, the majority of which were through predefined “categories”– such as compliant versus noncompliant, level of adherence, etc. Adherence was also reported quantitatively (e.g. fraction of medication used out of the amount that was prescribed, or number of adherent days per week in other studies [23,25,36,37,46]. 29 out of 36 studies reported adherence in “categories” (80.5%). Of these 29 studies, the number of categories differed: 2 categories (*n* = 17), 3 categories (*n* = 11), and 4 categories (*n* = 1).

Of the 17 studies that created 2 categories of adherence, definitions varied for what was “compliant” versus “noncompliant.” The threshold for good adherence and compliance varied between chelating at least 4 times a week [25], chelating at least 5 times a week [36,37,46], taking at least 80% of the recommended doses [32] or even taking at least 100% of the recommended to be compliant [31]. If the requirements were not met, patients in these studies were categorised as noncompliant.

Seven out of 36 studies reported “quantitative” adherence (19.4%). Of these 7 studies, adherence ranged from adherence ranged from 57% in one study [44] 9–98.4% compliance [43], with a median of 89.5% adherence.

### Predictors of adherence

A majority of the studies (*n* = 29) reported some findings of predictors or factors associated with the level of adherence, with many listing multiple, including: Type of iron chelation medication – DFP, DFX (both oral iron chelation medications), and DFO (parenteral iron chelation), etc. – (7/36, 19.4%), side effects/discomfort with medication (4/36, 11.1%), health related quality of life (2/36, 5.6%), psychological issues (4/36, 11.1%), personal issues, such as social issues, lack of family support, forgetfulness and tiredness (5/36, 13.9%), age (5/36, 13.9%), and length of treatment plan (1/36, 2.7%). On the other hand, some studies also reported some of these factors as actively not affecting adherence: one paper mentioned that having a family member with thalassaemia would not affect a patient's adherence level [20]. One study stated that ethnicity had no impact on adherence [32]. Use of oral iron chelators was associated with improved adherence in five studies [28,34,36,43,48]. One additional study found a higher adherence rate in patients who took oral chelators, however the relationship was not statistically significant [42].

### Adherence intervention

Five of the studies (13.9%) had a type of intervention component to them (Table 3). One of them examined the Medical Event Monitoring System to assess its strength as a measurement of compliant [39]. Both compared the system to patient diaries, and both found flaws in the MEMS measurement as a unique, but limited tool for evaluating adherence to medication [48]. This study called into question some of the more common ways of measuring adherence in thalassaemia patients, suggesting that the exact values and percentages gained from any study measuring adherence may need to be taken with precaution. The remaining four studies had designed interventions to improve adherence. Bahnasawy 2017 created a pharmacist centred intervention, where the clinical pharmacy gave regular calls to the patient about their regiment and found a significant improvement in drug-related problems and compliance [19]. Koch 1993 created a behavioural monitoring where patients were periodically rewarded for adherence during six months, and found that tracking and positive reinforcement were both associated with increased adherence [31]. Leonard 2017 used an ITP mobile app designed to improve disease knowledge and adherence, and patients tracked their own adherence over six months, again associated with positive improvements in adherence [35]. Lastly, Treadwell 2011 offered an in-person educational intervention, a day camp, designed to change perceptions and disease knowledge of ICT [44]. The camp did not yield positive results in changes in adherence, although patients reported being more informed about adherence. The authors suggested that ICT interventions should focus on family support or self-regulatory skills to cause changes in compliance.

**Table 3.** Description of adherence interventions.

Source (country)	Intervention purpose	Intervention description	Intervention results
Bahnasawy (2017) [19], Egypt	To determine the impact of clinical pharmacist-provided services on the outcome of iron-overloaded beta thalassaemia major children.	Regular phone calls and a patient-tailored medication chart detailed with drug dose, frequency and administration precautions were used to provide clinical pharmacy services	After 6 months of providing clinical pharmacy services to iron-overloaded paediatric BTM patient, there was a significant improvement in drug related problems, patient compliance to iron chelators, SF levels, patient healthcare satisfaction and HRQoL in the intervention group versus control.
Koch (1993) [31], USA	To determine the efficacy of a contingency and behavioural monitoring program designed to help patients increase or maintain their use of desferrioxamine	Behavioural intervention: patients were asked the number of days the wanted to achieve adherence and then asked to return a corresponding number of *via*ls every 2 weeks, with each week starting a new contract. Over the course of the 6 months, adherent patients would receive credit, which they could eventually accumulate over 10 visits to a gift of 20 dollars in value	Contingency tracking and positive reinforcement were related to increased compliance over the course of 6 months
Leonard (2017) [35], USA	To determine the feasibility of an “intensive training program” ITP mobile app, developed by the authors and its preliminary impact on adherence, disease knowledge, and health outcomes	Patient adherence was tracked using an ITP application, based on the pharmacy refill rates before enrolment and the rates for the 6 month enrolment period	Contingency tracking and positive reinforcement: Using a mobile ITP app with reminders was positively associated with ICT adherence
Olivieri (1991) [39], Canada	To determine the efficacy of MEMS (Medication Event Monitoring System)	MEMS: 88.7%, patients diaries reported a 95.7% compliance rate, patients often delayed *time* of medication, important as regular medication taking is needed	MEMS was considered a fairly good assessment, however would not be able to distinguish a missed dose from one taken doubled at the next bottle opening. (Incomplete understanding of *how* medications were being taken)
Treadwell (2011) [44], USA	To determine the value in an educational program in improving patient perceptions of ICT, and adherence	Desferal Day Camp– a camp that combines educational strategies with peer support	Patients reported satisfaction with interventions that focus on family support or self-regulatory skills. There were no changes in compliance

### Adherence related outcomes

#### Serum ferritin levels

The effects of adherence on serum ferritin levels were measured in 14 of the 36 studies (38.9%). All fifteen of these studies reported a drop in serum ferritin levels with increased adherence and compliance with iron chelators. Some found exceptions- Aina Mariana (2014) found that despite an overall trend of serum ferritin improvement with adherence, 21 patients still had serum ferritin levels above 1000 μg/L despite optimum adherence to either DFO, DFP, or DFX [14].

#### Other health outcomes

Outside of serum ferritin levels, adherence was shown to have a variety of physiological effects, notably impacting cardiac outcomes (4), liver health (2), psycho-neurological outcomes (2), and other hypothyroidism (1). Two studies also investigated the cost of thalassaemia medication and adherence: Payne 2008, conducted in the UK, estimated the mean weighted cost of medications in 3671 pounds, but that if individuals were 100% compliant, this cost should increase to 4421 pounds [41]. Zahed 2002 compared the cost of DFP and DFO, and found that deferiprone comes at significantly less of a cost to patients ($3000 per year) in comparison to desferrioxamine ($9000) [48].

## Discussion

### Principle findings

Adherence to iron chelation medication is a key component of health outcomes in thalassaemia patients due to the risks associated with iron overload. Yet there is little agreement on how to best measure iron adherence, or what levels of adherence are appropriate for optimal treatment outcomes. The objectives of this systematic review were to identify global values of adherence to iron chelation therapy and the factors associated with good or poor adherence as well as survey commonly employed methods of assessing adherence in paediatric populations.

We identified 36 studies that met our pre-set criteria. Of these studies, the majority were clinical trials that assessed patient adherence through a variety of methods including: parent reports, patient reports, provider reporting, chart review, prescription refill rate, and medication logs.

Due to the wide variety in how each study both defined and reported adherence, both categorically and quantitatively, it was difficult to compare the results of each study. Amongst categorical studies, adherence was divided into 2–4 groups. The majority (17) of these studies divided patients into two groups “compliant versus noncompliant”, or “good versus poor adherence”. However, the actual definitions of compliance in these studies were different greatly, with some defining compliance based on the number of pills taken, others based on the number of days a medication was taken, while others based on questionnaires. Even when the same marker of adherence was used, such as the number of days medication was taken per week, the threshold for the number of days considered “compliant” would vary from study to study,

Only 7 studies measured adherence quantitatively, based on the percentages of doses taken. Nevertheless, there was still a wide range of reported adherence, from the lowest reported adherence of 57% to the highest reported adherence of 88.4%. Again, this variance could be attributed to the different methodologies used to assess adherence from patient self-report to physician report to pill count. In comparison to a more objective measurement such as pill count via MEMS system, patient self-report may lead to more inaccuracies, with physician/provider rating, likely lending itself to the biases of the physician and second-hand account of adherence history. In addition, some studies chose to focus on populations with comorbidities [49]. This would lead to a Berkson’s selection bias and likely underestimate the value of true adherence in the general population. Thus, while studies had very different reports on adherence, it is hard to understand whether this heterogeneity is due to measurement, definition, or scale. Future research may centre on standardizing adherence reporting (e.g. using a common questionnaire such as MMAS for all patient self-reports.) One study in particular examined the Medical Event Monitoring System as a potential standard for adherence measurement, but found flaws in using it as a single, limited tool in evaluating adherence, suggesting a need for further investigation into the validity of different adherence reporting systems before selecting a standard measurement for investigative purposes [39].

With regards to developing a universal adherence evaluation, it may be difficult to discern which of these evaluative tools is most useful. Patient self-report is commonly used due to efficiency, low cost, and easy implementation [9,49,50]. Patient-reported medication adherence measures additionally benefit from patient input into medication-taking behaviours (e.g. taking medication as prescribed, refilling prescriptions on schedule), barriers to medication adherence (e.g. adverse side effects), and beliefs associated with medication adherence (e.g. necessity of medication). Alternatively, medication and prescription monitoring systems do not allow for this commentary, but may be less prone to bias or forgetfulness in reporting. Both qualitative and quantitative adherence presented challenges due to differences in methodologies in assessing adherence. However, qualitative adherence, while more common among these studies, provides an additional barrier in comparing adherence rates due to different definitions and categorizations of adherence and non-adherence. Ultimately, developing a standard for quantitative adherence measurement may prove superior as a means for universal adherence evaluation and comparison in future research.

That said, other study results were more consistent across papers–predictors associated with iron chelation adherence, included type of iron chelation medication (7), side effects (4), personal issues (social, familial etc.) (5), age (5), psychological issues (4), and health-related quality of life (2). The effect of both the type of iron chelation medication and medication side effects within these studies again reinforces that the different side effect profiles, route of administration, and regimen of these chelators varies significantly and can play a powerful role in adherence for patients. Such studies highlight the importance of discussing these factors with patients when choosing a medication. The impact of psychological and personal well-being on adherence in paediatric patients specifically may be due to the suggests that adherence should be approached with a multidisciplinary approach in paediatric patients, and the importance of screening paediatric patients with low adherence for home safety, resources, and psychological well-being. Use of oral iron chelators was associated with improved adherence in five studies [28,34,36,43,48], with one additional study also finding non-statistically significant rates of higher adherence with oral chelators [42]. This relationship is most likely attributed to the ease with which patients could take these chelators, and the lower perceived side effects.

The strongest effect of adherence appears to be in serum ferritin levels; all 14 studies which examined these levels found a drop in serum ferritin levels with increased adherence to an iron chelator, indicating the effectiveness of the medication. Fewer studies examined other outcomes of iron chelation adherence, although the biggest effect examined was cardiac outcomes. This may have been because some of the outcomes of adherence in the paediatric population may not present until these patients are adults. Improving adherence has a clear and consistent relationship with cardiac outcomes across the studies which examined it, but the important effects of adherence on liver health and endocrine outcomes should also be considered and further studied. Two studies examined the cost to patients and families of the medication, the impact of these costs on patients, as both a factor in adherence as well as health-related quality of life could also be further studied. In addition, the top outcomes associated with higher adherence in adult patients included lower serum ferritin levels, and fewer adverse cardiac, liver, and psychoneurological outcome outcomes.

Of the papers that focussed on an intervention, four studies had designed interventions to improve adherence (the fifth was the Olivieri 1991 paper on MEMS) [39]. Each study had a unique strategy to improve adherence– calling the patient (Bahnasawy 2017), reward systems (Koch 1993), and mobile apps (Leonard 2017) all were associated with increased adherence in their respective studies [19,31,35]. Bahnasawy 2017 studied a pharmacist centred intervention, where the clinical pharmacy gave regular calls to the patient about their regiment; and found a significant improvement in compliance [19]. Such interventions underscore the role of care members in supporting patients and caregivers in understanding the importance of adhering to therapy by making information available, raising awareness, providing age-appropriate education, and using techniques such as shared decision making and motivational interviewing. However, short term educational day camp intervention (Treadwell 2011), reported an increased understanding of adherence, but no significant changes in the level of adherence [44]. Reflecting on the methods that were successful in changing adherence rates, consistent, long-term engagement with patients, regardless of its form, seemed crucial in bolstering adherence. Other long-term interventions, or modifications upon the studies above would be an important route to investigating the strength of ICT adherence interventions in clinical practice.

### Strengths

Our systematic review had many strengths. We followed the recommended methodology for rigorous systematic reviews [51]. Moreover, we conducted the review with a highly sensitive search strategy guided by a librarian information specialist with no language restrictions to minimize publication bias and identify the largest possible number of relevant studies. Furthermore, the search included published systematic reviews, clinical trial registries, and various electronic databases. Although our search was limited to studies published since 1980, this historically dates to when deferoxamine and other iron chelators began being used more widely, so it is likely that a majority of the literature related to this topic was found. We also were able to find a relatively large portion of the existing literature that met our inclusion and exclusion criteria (36 out of 522).

### Limitations

There are some potential limitations of our systematic review that warrant discussion. Like any systematic literature review, despite our broad and comprehensive search criteria, the possibility of missing a few relevant articles cannot be excluded. Additionally, the study sample size and age, the definition of adherence, and measurement of adherence varied, prohibiting a meta-analysis from being performed. Furthermore, this study was limited to peer-reviewed journals. As such, there may be a potential bias in the significance of the results of the published data.

## Conclusions

In conclusion, due to a lack of clinical consensus on what adherence is and how it is defined, it is difficult to compare the adherence reporting of different studies. However, the patterns that do emerge from these studies suggest that certain factors are consistently correlated with adherence in paediatric populations, including the type of medications used, side effects, perception and understanding of the patient, and other personal factors, suggesting that intervention in this stage of thalassaemia patient treatment and increased dialogue between physicians and their patients about their perception and side-effects of their medication could be key to long term health outcomes, specifically cardiac outcomes, as noted above. Further research on adherence with rigorous research designs is needed to understand the factors contributing to and affected by adherence and improve clinical consensus on adherence. These research efforts will be crucial in informing evidence-based interventions to improve adherence and health outcomes in thalassaemia patients.

## Data Availability

The authors confirm that the data supporting the findings of this study are available within the article and from all included articles in this systematic review. Additional summary data that support the findings of this study are available from the corresponding author, [SMB], upon reasonable request.
